# Diabetic Ephrin-B2-Stimulated Peripheral Blood Mononuclear Cells Enhance Poststroke Recovery in Mice

**DOI:** 10.1155/2018/2431567

**Published:** 2018-03-15

**Authors:** Rose Hilal, Marine Poittevin, Adrien Pasteur-Rousseau, Adrien Cogo, Gabrielle Mangin, Marie Chevauché, Yasmine Ziat, José Vilar, Jean-Marie Launay, Jean-François Gautier, Dong Broquères-You, Bernard I. Levy, Tatyana Merkulova-Rainon, Nathalie Kubis

**Affiliations:** ^1^Sorbonne Paris Cité, CART, INSERM U965, Université Paris Diderot, 75475 Paris, France; ^2^Service de Physiologie Clinique, AP-HP, Hôpital Lariboisière, 75475 Paris, France; ^3^INSERM U970, Paris Cardiovascular Research Center, 75737 Paris, France; ^4^Service de Biochimie et Biologie Moléculaire, AP-HP, Hôpital Lariboisière, 75475 Paris, France; ^5^CUDC, AP-HP, Hôpital Lariboisière, 75475 Paris, France; ^6^Institut Vaisseaux Sang, Hôpital Lariboisière, 75475 Paris, France

## Abstract

Clinical trials of cell therapy in stroke favor autologous cell transplantation. To date, feasibility studies have used bone marrow-derived mononuclear cells, but harvesting bone marrow cells is invasive thus complicating bedside treatment. We investigated the therapeutic potential of peripheral blood-derived mononuclear cells (PB-MNC) harvested from diabetic patients and stimulated by ephrin-B2 (PB-MNC+) (500,000 cells), injected intravenously 18–24 hours after induced cerebral ischemia in mice. Infarct volume, neurological deficit, neurogenesis, angiogenesis, and inflammation were investigated as were the potential mechanisms of PB-MNC+ cells in poststroke neurorepair. At D3, infarct volume was reduced by 60% and 49% compared to unstimulated PB-MNC and PBS-treated mice, respectively. Compared to PBS, injection of PB-MNC+ increased cell proliferation in the peri-infarct area and the subventricular zone, decreased microglia/macrophage cell density, and upregulated TGF-*β* expression. At D14, microvessel density was decreased and functional recovery was enhanced compared to PBS-treated mice, whereas plasma levels of BDNF, a major regulator of neuroplasticity, were increased in mice treated with PB-MNC+ compared to the other two groups. Cell transcriptional analysis showed that ephrin-B2 induced phenotype switching of PB-MNC by upregulating genes controlling cell proliferation, inflammation, and angiogenesis, as confirmed by adhesion and Matrigel assays. *Conclusions*. This feasibility study suggests that PB-MNC+ transplantation poststroke could be a promising approach but warrants further investigation. If confirmed, this rapid, noninvasive bedside cell therapy strategy could be applied to stroke patients at the acute phase.

## 1. Introduction

Providing appropriate treatment to all stroke patients remains a genuine challenge. Sixteen million strokes occur every year [1], one every four minutes. Of these, 25% of patients die, 40% remain disabled, and 25% develop dementia. Stroke represents a considerable economic burden, with costs reaching 8.3 billion euros per year in France (Regional Health Agency report, 2013). Diabetes is a known independent risk factor for stroke, increasing its incidence threefold and worsening both the severity of the event and stroke-related mortality [2]. These observations are all the more alarming considering the anticipated doubling of the prevalence of diabetes by 2030 (World Health Organization, 2012).

Early recanalization following proximal cerebral artery occlusion is critical; stent retriever thrombectomy combined with recombinant tissue plasminogen activator (rtPA [Alteplase]) reduces disability at 90 days and improves functional independence when performed within 8 hours poststroke [3]. Nevertheless, the limited therapeutic time window and the scarcity of interventional neuroradiology centers mean that very few patients actually receive effective treatment. The search for strategies to extend the limited therapeutic window is challenging, particularly those focusing on restoring neurological damage.

Cell therapy-based approaches hold considerable promise. Translational research favors transplantation of autologous cells from various tissues and organs, in a more or less differentiated state. Expansion of mesenchymal stem cells requires several weeks of culture processing. In contrast, bone marrow-derived mononuclear cells (BM-MNC) can be obtained simply by density gradient centrifugation rendering them immediately ready for use. BM-MNC transplantation improves poststroke neurological deficit in rodents [4], and clinical trials have shown the feasibility of local [5], intra-arterial [6, 7], or intravenous [8, 9] administration. The underlying mechanisms are not fully understood: the transplanted cells show rare differentiation into endothelial or neural cells [10], but they do contribute to brain repair processes by stimulating endogenous angiogenesis [11], which in turn induces neurogenesis [12] and modulates the inflammatory response [13], thus counteracting the inflammatory cascade that creates a hostile environment compromising the survival of new cells.

Peripheral blood-derived MNC (PB-MNC) transplantation may be an interesting alternative because unlike BM-MNC, PB-MNC can be easily collected at the bedside. Furthermore, in a mouse model of hind limb ischemia, we previously showed that pretreating PB-MNC with ephrin-B2 considerably improves their therapeutic potential, especially when isolated from diabetic patients [14]. Ephrin-B2 belongs to the Eph/ephrin family of membrane-bound cell signaling molecules that are essential for embryonic development of the nervous system and vasculature and for neurogenesis and angiogenesis in adults [15, 16]. In addition, both ephrin-B2 and its receptor EphB4 are expressed in mature monocytes and lymphocytes and regulate their motility, adhesion, transmigration, and the secretion of inflammatory mediators [17]. These data suggest that activating the ephrin-B2/EphB4 pathway of mononuclear cells may provide notable therapeutic benefits in stroke.

We therefore set out to evaluate the therapeutic potential of PB-MNC isolated from diabetic patients (a population at risk for stroke) and stimulated (PB-MNC+) or not by ephrin-B2, administered intravenously into healthy, nondiabetic, adult male C57Bl6J mice subjected to focal cerebral ischemia. Our primary objective was to assess infarct volume and neurological deficits in mice poststroke; the secondary objective was to assess whether peripheral blood transplanted cells (PB-MNC or PB-MNC+) also affected poststroke angiogenesis, inflammatory response, and tissue repair, as described for BM-MNC. Lastly, we investigated the in vitro properties of PB-MNC+.

## 2. Materials and Methods

### 2.1. PB-MNC Cells and Ephrin-B2/Fc Binding

Blood samples from type-2 diabetic patients from the CUDC (Centre Universitaire du Diabète et de ses Complications) Department of the Lariboisière Hospital (Paris) were collected in heparinized tubes, during the patients' routine annual check-up. All patients gave written informed consent. Mean age of the cell donors was 62 ± 8 years old and 61% were males with known diabetes for a duration of 14 ± 8 years; 73% had hypertension, 51% had dyslipidemia, 24% were current smokers, and 21% were former smokers. In addition to oral antidiabetic drugs (gliclazide, glibenclamide, metformin, glimepiride, exenatide), donors were on hypocholesterolemic drugs (rosuvastatin, atorvastatin, ezetimibe), antiplatelet therapy, and antihypertensive drugs (calcium channel blockers, angiotensin II receptor blockers, beta-blockers, angiotensin-converting enzyme inhibitors, and thiazide diuretics).

PB-MNC were isolated by density gradient centrifugation using Pancoll (PAN Biotech, Aidenbach, Germany). Cells were washed with PBS and incubated in M199 medium with or without 15 *μ*g/mL of recombinant ephrin-B2/Fc (R&D systems, Minneapolis, MN) at 37°C for 30 minutes. Cell viability was assessed using the Muse Count & Viability Kit and a Muse Cell Analyzer (Millipore Corporation, Hayward, CA) and was determined as >96%. Optimal stimulation conditions have been described previously [14].

The subcellular composition of PB-MNC transplants and the PB-MNC cellular ephrin-B2/Fc-binding subsets were determined using flow cytometry analysis as previously described [14]. Briefly, to identify cellular subpopulations, we incubated the cells (30 min at 4°C) with a mixture of directly conjugated antibodies specific for the following surface antigens: V450-antihuman CD3, PerCP-Cy5.5-antihuman CD4, FITC-antihuman CD8, APC-antihuman CD19, APC-H7-antihuman CD14, V500-antihuman-CD45, and a PE-Cy7-antihuman CD34 (all from BD Biosciences). The cells were then washed twice with PBS and analyzed using a LSRII flow cytometer (BD Biosciences). The percentage of positive cells was determined using the FlowJo software (Tree Star, Ashland, OR). In a series of similar experiments, PB-MNC preincubated with biotinylated ephrin-B2/Fc were incubated with the above mixture of surface antigen-specific antibodies supplemented with PE-streptavidin (BD Biosciences) to determine the percentage of ephrin-B2-binding cells within each PB-MNC cellular subset.

### 2.2. Animal and Group Distribution

All animal experiments and surgical procedures were performed in accordance with the European Community Directive (2010/63/EU), the ARRIVE (Animal Research Reporting In Vivo Experiments) guidelines, and the French national guidelines for the care and use of laboratory animals. The study was approved by the Local Ethics Committee in Animal Experimentation (protocol number CEEALV/2012-11-02).

**Figure 1:**
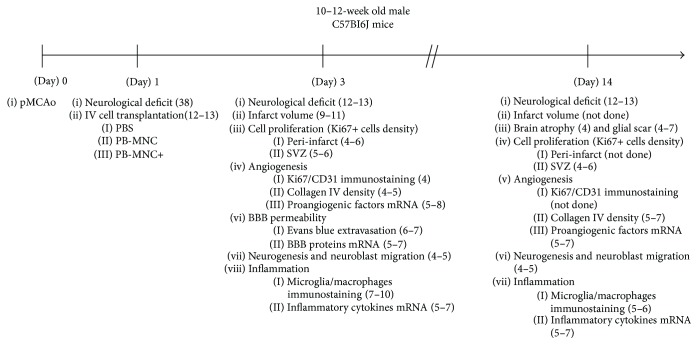
Experimental protocol and time schedule. pMCAo: permanent middle cerebral artery occlusion; IV: intravenous; PBS: phosphate-buffered saline; PB-MNC: peripheral blood mononuclear cells; PB-MNC+: peripheral blood mononuclear cells stimulated by ephrin-B2; BBB: blood-brain barrier; SVZ: subventricular zone. There were 3 groups for each endpoint (number of mice/group). Infarct volume was not assessed at D14 because the infarct was barely detectable. Peri-infarct cell proliferation and angiogenesis evaluated on Ki67+/CD31+ double-positive cell density were not performed at D14, because of the scarce number of Ki67+ cells in the peri-infarct area at D14.

Experiments were performed on male adult C57/BL6 mice (age, 10–12 weeks; 20–25 g) (Janvier Labs, Le Genest-Saint-Isle, France). Focal cerebral ischemia was induced at D0 and intravenous injection of PBS, PB-MNC, or PB-MNC+ performed on D1. Poststroke neurological deficit was first assessed on D1 before randomization to cell transplantation and then at D3 and D14 to assess differential outcome between groups. Infarct volume and blood-brain barrier (BBB) permeability were evaluated at D3, and angiogenesis, neurogenesis, and inflammation were evaluated at D3 and D14 (Figure 1). Microarray analyses were performed on PB-MNC and PB-MNC+, and in vitro properties of these cells were assessed on adhesion assays and capillary-like tube formation assays on Matrigel.

The examiner was blinded to all scoring and data analyses to prevent sample identification (phosphate-buffered saline- (PBS-) treated control mice, PB-MNC, or PB-MNC+ transplantation). Codes were attributed to each mouse by an independent team member.

The sample size calculation was not possible a priori (new feasibility and mechanistic study), but based upon the experience of the team, given the surgery procedure and the outcome assessments.

### 2.3. Focal Cerebral Ischemia and Intravenous PB-MNC Administration

Focal cerebral ischemia was induced by electrocoagulation of the left middle cerebral artery leading to permanent occlusion (pMCAo), as previously described [18]. Between 18 and 24 hours after pMCAo, animals were randomized to intravenous PBS, PB-MNC, or PB-MNC+. Stratified randomization was achieved by assigning each mouse to a group according to the degree of neurological impairment. Cells were then injected via the retro orbital vein (5 × 10^5^ cells suspended in 200 *μ*L of PBS per mouse, equivalent to 25 million cells/kg). The dose of cells injected was determined on the basis of the dose-effect relationships established previously in a mouse model of hind limb ischemia [14]. A mix of different patients' blood samples was administered to the mice. For the surgical procedure, mice were anesthetized using isoflurane (initially 2%, followed by 1.5 to 1.8% in O_2_). Intraperitoneal buprenorphine (0.1 mg/kg) was administered 30 minutes before starting anesthesia and repeated every 12 hours for 48 hours. No immunosuppressants were used.

### 2.4. Neurological Deficit Assessment

Neurological deficit was assessed at D1 and pMCAo mice were compared to nonoperated mice, before randomization into the three groups. Following randomization to PBS, PB-MNC, or PB-MNC+, neurological deficit was assessed at D3 and D14 by calculating a global neurological score (/38): the lower the score, the more severe the deficit, as based on six neurological tests (neurological score, circle test, grip and string tests, beam walking, and pole test) [18]. In addition, at the same time points, the two hind paws were immersed in a water-soluble, nontoxic liquid ink. Mice were allowed to walk freely toward a bright box, along a dark 8/3/100 cm corridor lined with paper. Each mouse performed the test three times, and the best track was recorded. Results are expressed as the toe spread, determined by calculating the distance between the first and fifth toes, the smallest value indicating the greatest deficit [19].

### 2.5. Infarct Volume and Brain Atrophy Assessment

Infarct volume was assessed three days after pMCAo, on coronal 30 *μ*m sections stained with cresyl violet. The cortical infarct area was measured on every eighth section (30 *μ*m thick) and volume (mm^3^) calculated using ImageJ software (National Institutes of Health, Bethesda, MD) [18]. Brain atrophy was assessed at day 14, as follows: the total brain volume (left hemisphere + right hemisphere, excluding bulbus olfactorius, cerebellum, and brainstem) and the size of the lateral ventricles (left ventricle + right ventricle) were measured by manually drawing respective regions of interest on every 8th coronal section integrating the distance between sections and the thickness of each section using NIH ImageJ (v 1.33, National Institute of Health, USA) analysis software. Brain matter was then calculated by subtraction of ventricle volume from total brain volume. Data are expressed as arbitrary units (a.u.).

### 2.6. Immunohistochemistry and Morphological Analysis

Coronal 30 *μ*m free-floating sections were incubated with primary antibody overnight at 4°C. Anti-Ki67 (1 : 200, Abcam, UK), antidoublecortin (DCX) (1 : 100, Santa Cruz, USA), anticollagen IV (1 : 200, Abcam, UK), anti-CD31 (1 : 200, BD Biosciences, USA), anti-Iba1 (1 : 200, Wako, Japan), and anti-GFAP (1 : 400, Millipore, Burlington, USA) antibodies were used to detect proliferative cells, neuroblasts, basal lamina of endothelial cells, endothelial cells, microglia/macrophage cells, and astrocytes, respectively. Appropriate Alexa Fluor 594- or 488-labeled secondary antibodies (1 : 400, Molecular Probes, Eugene, OR) were applied for one hour at room temperature. Specificity was checked by omitting the primary antibody.

Cell counts and staining density measurements were performed at three coronal brain levels (+0.80, −0.80, and −1.20 mm relative to bregma), all consistent within the infarct area. Cell proliferation was evaluated in the peri-infarct area and in the subventricular zone (SVZ) and expressed as the mean number of Ki67+ cells in each area. Angiogenesis was assessed in three regions of interest (0.06 mm^2^ each) located in the peri-infarct area and expressed as the average number of Ki67+/CD31+ cells per region of interest. Microvessel density was evaluated after collagen IV immunolabeling by calculating the integrated pixel density of collagen IV immunolabeling per total microscopic field (20x), using NIH ImageJ software. Microglia/macrophage density was evaluated in the peri-infarct area as described above after Iba-1 immunolabeling. Glial scar was evaluated in the peri-infarct area after astrocyte immunolabeling and quantification performed as described above for angiogenesis. Neurogenesis was evaluated in the whole section by assessing neuroblast density after DCX immunolabeling. Neuroblast (DCX+) migration was calculated by measuring the distance from the SVZ to the cell cluster nearest to the ischemic area. The maximal distance that SVZ cells covered (*μ*m) was measured and the mean value calculated between the three sections.

### 2.7. Evaluation of Blood-Brain Barrier Permeability

BBB permeability was assessed three days poststroke, as previously described [12].

### 2.8. Quantitative Real-Time Analysis of mRNAs (qRT-PCR) for BBB Components, Angiogenesis, and Inflammatory Cytokines

Total RNA was isolated from ipsilateral hemispheres of ischemic brains using the RNeasy Lipid Mini Kit (Qiagen, Hilden, Germany) according to standard procedures. Reverse transcription was performed with Ready-to-Go RT-PCR Beads (GE Healthcare, Uppsala, Sweden) and real-time PCR was performed using a Mesa Green qPCR kit (Eurogentec, Angers, France) on a Mastercycler Realplex [18] (Eppendorf AG, Hamburg, Germany). We used custom-designed primers for all genes of interest (Table 1) and a ready-to-use primer for the housekeeping peptidylpropyl isomerase A (cyclophilin A) (Qiagen, Courtaboeuf, France). We ran all assays in triplicate. We normalized the results for each individual gene to the level of the housekeeping gene encoding cyclophilin A. Results are expressed as arbitrary units (a.u.).

### 2.9. Plasma Cytokine Measurements

Fourteen days of poststroke, blood samples were collected in heparinized microtubes, centrifuged at 4°C, and plasma samples were collected. Vascular endothelial growth factor-A (VEGF-A) and brain-derived neurotrophic factor (BDNF) (R&D systems, France) levels were detected by ELISA according to manufacturer's protocols.

### 2.10. Microarray

PB-MNC isolated from seven type-2 diabetic patients and stimulated or not with ephrin-B2 were incubated in M199 medium for one hour or four hours and then processed to determine differential gene expression. At the end of incubation, cells were collected and total RNA was isolated using NucleoSpin® RNA (Macherey-Nagel GmbH & Co. KG, Düren, Germany). Gene expression analysis of PB-MNC was performed by the Genomics Services Unit of MACSmolecular (Miltenyi Biotec GmbH, Bergisch Gladbach, Germany) using the Agilent Whole Human Genome Oligo Microarrays, dual color (Agilent Technologies, Santa Clara, CA, USA). RNA from unstimulated and stimulated cells from the same patient was, respectively, Cy3- and Cy5-labeled using the Agilent Low Input Quick Amp Labeling Kit (Agilent Technologies). The corresponding cRNA were combined and hybridized overnight to Agilent Whole Human Genome Oligo Microarrays 4×44K using the Agilent Gene Expression Hybridization Kit, according to the Agilent 60-mer oligo microarray processing protocol (Agilent Technologies). Fluorescence signals of the hybridized Agilent Oligo Microarrays were detected using the Agilent DNA microarray scanner. The Agilent Feature Extraction Software (FES) was used to read and process the microarray image files, including determination of feature intensities and ratios with background subtraction and normalization, rejection of outliers, and calculation of statistical confidence intervals (*P* values). The FES-derived output data files were further analyzed using the Rosetta Resolver gene expression data analysis system (Rosetta Biosoftware, Seattle, WA, USA) to determine the differential gene expression.

### 2.11. In Vitro Adhesion Assay

Human umbilical vein endothelial cells (HUVEC) were isolated and cultured as previously described [20]. Two days before isolating PB-MNC, HUVEC were seeded in a 0.2% gelatin-coated 96-well plate at 10^4^ cells/well. PB-MNC were obtained from ten patients and labeled with a Vybrant CFDA-SE Cell Tracer (Molecular Probes, Eugene, OR, USA) according to manufacturer's recommendations; PB-MNC were stimulated or not with ephrin-B2/Fc and added to a HUVEC monolayer at 7.5 × 10^4^ cells/well. After incubation for one hour at 37°C, the percentage of adherent PB-MNC was determined using a Victor3 spectrofluorimeter (PerkinElmer, Turku, Finland).

### 2.12. Capillary-Like Tube Formation Assay on Matrigel

Cells were obtained from seven patients and cocultured with HUVEC on Matrigel to allow the formation of a capillary-like network. HUVEC and PB-MNC were seeded on the preformed Matrigel gel in a *μ*-Slide Angiogenesis ibiTreat Microscopy Chamber (Ibidi, Martinsried, Germany) at 7 × 10^3^ cells/well. After culture at 37°C for 24 hours in a humidified atmosphere containing 5% CO_2_, capillary-like structures were observed with an Axio Observer.Z1 fluorescence microscope (Carl Zeiss MicroImaging GmbH, Göttingen, Germany). Images were taken using a Baumer TXD14 digital monochrome progressive scan camera (Baumer Optronic GmbH, Radeberg, Germany) and Archimed 4.7.0 software (Microvision Instruments, France). The number of branch points of the capillary tube formation per microscopic field was quantified using HistoLab software (Microvision Instruments).

### 2.13. Cell Tracking

At 1 hour, 1 day, 3 days, and 14 days after cell transplantation, ipsilateral and contralateral hemispheres, lungs, spleen, liver, and kidneys were removed and snap frozen in liquid nitrogen, after flushing the brain vasculature. The presence of human cells was detected using human genomic DNA polymerase chain reaction (PCR). DNA samples (100 ng per reaction) were subjected to PCR to detect a 1171 bp fragment of the human chromosome 17-specific satellite region [12]. P17H8 sequence was amplified using the Light Cycler 1.5 Instrument (Roche Diagnostics), the Light Cycler FastStart DNA Master SYBR Green I, and the following primers: forward, 5′-ACACTCTTTTTGCAGGATCTA-3′ and reverse, 5′-AGCAATGTGAAACTCTGGGA-3′. As a positive control, 0.5 pg of human genomic DNA isolated from PB-MNC from the patients (0.0005% of mouse DNA, low detection limit) was added to the fourth replicate of each sample. As a negative control, the template was replaced by PCR-grade water. For quantification, standard curves were generated by serial dilution of a human genomic DNA in the presence of 100 ng of mouse genomic DNA isolated from the liver or from the tibialis anterior muscle. Specificity of the amplified PCR product was assessed by performing a melting curve analysis (the specific p17H8 product melts at 84°C). PCR products were also analyzed by 1.5% agarose gel electrophoresis to determine the presence of P17H8-specific band of 1171 bp.

### 2.14. Statistical Analysis

Statistical analyses were performed with Prism 5 Software (Prism v5.03; GraphPad, San Diego, CA). Data are expressed as mean ± SD. A paired *t*-test was used to compare HUVEC properties when PB-MNC or PB-MNC+ were added to the HUVEC monolayer. Data comparisons between the three groups were performed using the Kruskal-Wallis test with a post hoc Dunn's test. Comparisons of neurogenesis, angiogenesis, and inflammation between D3 and D14 in each group were made with an unpaired *t*-test. The sample size was based on the low operative mortality; if secondary hemorrhage occurred during procedure, mice were excluded from the analysis. Statistical significance was set at *P* < 0.05.

## 3. Results

### 3.1. Survival and Neurological Deficit

**Figure 2:**
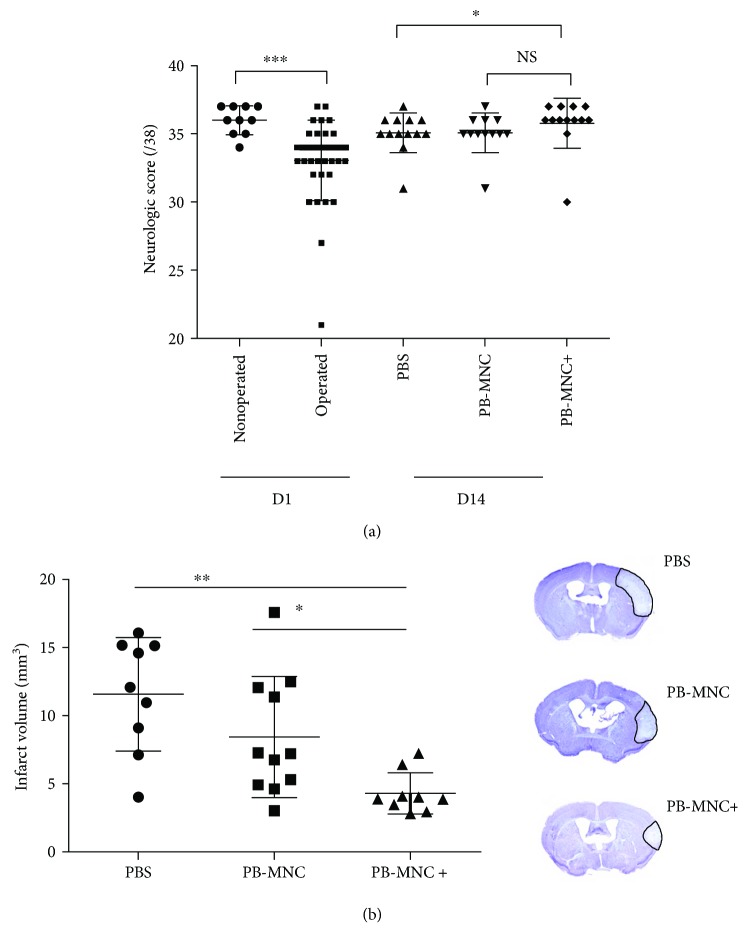
Neurological score is increased whereas infarct volume is decreased in mice engrafted with ephrin-B2-stimulated diabetic PB-MNC. (a) At D1, there was a significant reduction in the neurological score of operated versus nonoperated mice (*N* = 36). At D14, a significant increase in the neurological score was detected in the PB-MNC+ versus the PBS group but not versus the PB-MNC group (*N* = 12–13/group). (b) At D3, there was a significant reduction in infarct volume in PB-MNC+ versus PBS and PB-MNC-treated mice (*N* = 9–11/group). ^∗^*P* < 0.05; ^∗∗^*P* < 0.01; ^∗∗∗^*P* < 0.001.

No mice died during the two-week follow-up, regardless of the transplant group. At D1, we observed a significant reduction in the neurological score in operated (pMCAo) compared to nonoperated mice (33.1 ± 3.0 versus 36 ± 1.0, resp., *P* < 0.001). Operated mice were then randomized to PB-MNC+, PB-MNC, or PBS. At D3, neurological scores increased in all three groups compared to D1, though the differences between PB-MNC+, PB-MNC, and PBS were not significant (35.86 ± 0.26, 36.29 ± 0.36, and 35.00 ± 0.77, resp.). At D14, the neurological score of the PB-MNC+ group was significantly higher than that of the PBS group (35.8 ± 1.8 versus 35.1 ± 1.4; *P* < 0.05); the difference between the PB-MNC+ group and the PB-MNC group was however not significant (35.1 ± 1.4) (*N* = 12–13) (Figure 2(a)). No significant intergroup differences were observed on the ink test assessment, regardless of the time point (data not shown).

### 3.2. Infarct Volume, Brain Atrophy, and Glial Scar

At D3, we observed a significant reduction in infarct volume in mice treated with PB-MNC+ (4.3 ± 1.5 mm^3^) compared to those receiving PB-MNC (8.4 ± 4.4 mm^3^, *P* < 0.05) or PBS (11.6 ± 4.2 mm^3^, *P* < 0.01) (*P* = 0.0017) (*N* = 9–11) (Figure 2(b)). There was no hemorrhagic transformation.

At D14, infarct volume in all groups was barely detectable and was therefore not measured.

Brain atrophy calculation was not statistically significantly different between PB-MNC+ (124.7 ± 2.9 a.u.), PB-MNC (135.4 ± 8.8 a.u.), and PBS groups (128.6 ± 9.4 a.u.) (*N* = 4). Glial cell density was not different between PB-MNC+ (39.1 ± 5.8 cells), PB-MNC (40.7 ± 4.7 cells), or PBS (43.6 ± 6.8 cells) (*N* = 4–7).

### 3.3. Cell Proliferation

**Figure 3:**
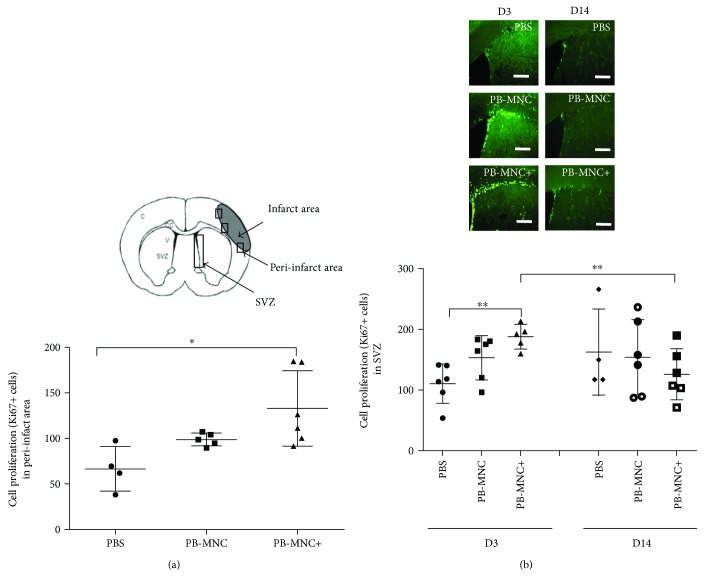
Ephrin-B2-stimulated diabetic PB-MNC promote cell proliferation poststroke. (a) In the peri-infarct area at D3, Ki67+ cell numbers were significantly greater in the PB-MNC+ group versus the PBS group, but there were no significant differences between the PB-MNC group and the other two groups (*N* = 4–6/group). (b) In the subventricular zone at D3, there was a significant increase in Ki67+ cells in PB-MNC+-treated mice compared to PBS-treated mice (*N* = 5–6/group). By D14, no significant differences were evidenced between PB-MNC+, PB-MNC, and PBS (*N* = 4–6/group). There were no significant differences between D3 and D14 in the PBS and PB-MNC groups, whereas there were significantly fewer Ki67+ cells in the PB-MNC+ group. Data are presented as mean ± SD. ^∗^*P* < 0.05; ^∗∗^*P* < 0.01. Scale bar = 200 *μ*m.

At D3, Ki67+ cell levels in the peri-infarct area were significantly increased in the PB-MNC+ group compared to the PBS group (132.9 ± 41.3 cells versus 66.6 ± 24.5 cells, resp.; *P* < 0.05), but there were no significant differences between the number of Ki67+ cells in the PB-MNC group (98.8 ± 7.0 cells) and the two other groups (*N* = 4–6) (Figure 3(a)). In the subventricular zone (SVZ), there was also a significant increase in Ki67+ cells in mice treated by PB-MNC+ compared to PBS-treated mice (187.6 ± 20.2 cells versus 110.4 ± 32.6 cells; *P* < 0.01), but no significant difference between Ki67+ cells in PB-MNC-treated mice (153.0 ± 36.2) and PBS-treated mice (*N* = 5–6) (Figure 3(b)).

At D14, very few Ki67+ cells were present in the peri-infarct area of mice in all three groups. In the SVZ, although Ki67+ cells continued to proliferate, differences between the PB-MNC+, PB-MNC, and PBS groups were no longer significant (125.7 ± 42.0, 153.8 ± 61.7, and 162.3 ± 70.5 cells, resp.) (*N* = 4–6) (Figure 3(b)). By D14, there were no significant differences in Ki67+ cell numbers in the SVZ compared to D3 in the PBS and PB-MNC groups, whereas this parameter was significantly decreased in the PB-MNC+ group (*P* = 0.017) (Figure 3(b)).

### 3.4. Angiogenesis

Angiogenesis was assessed by double immunolabeled Ki67+/CD31+ cell counts, microvessel density after collagen IV immunostaining, and levels of mRNA encoding proangiogenic proteins.

**Figure 4:**
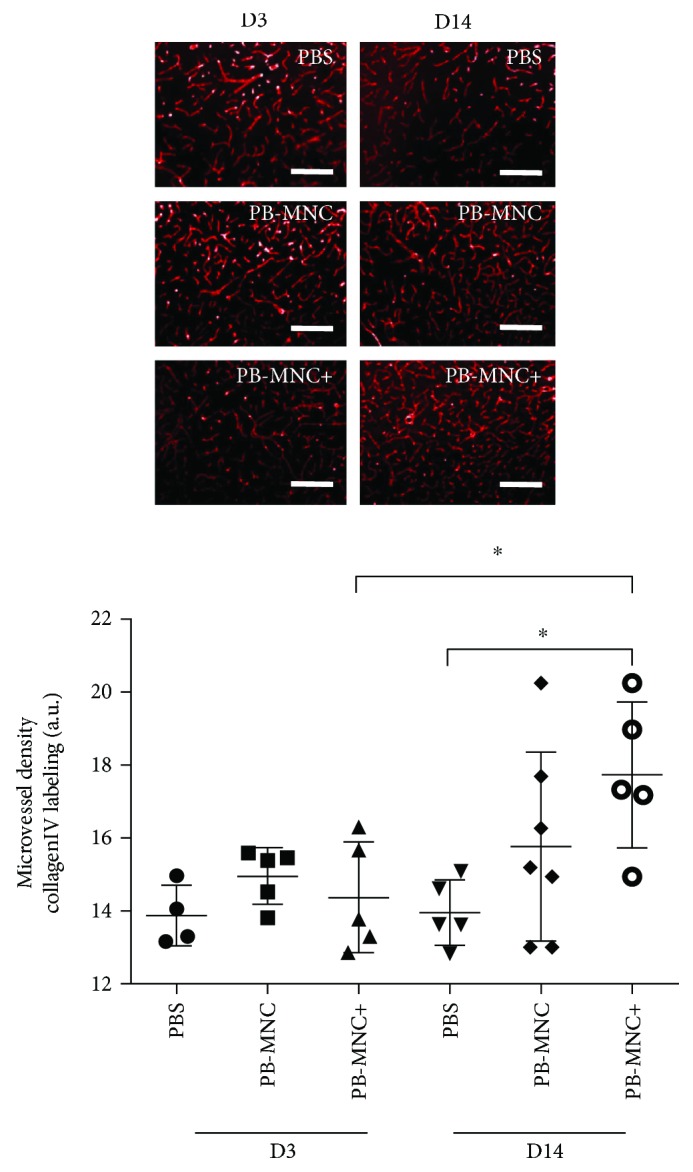
Ephrin-B2-stimulated diabetic PB-MNC mice significantly increase angiogenesis in the peri-infarct area. Although microvessel density on D3 was not significantly different between the PB-MNC+, PB-MNC, and PBS groups (*N* = 4–5/group), it was significantly greater on D14 in the PB-MNC+ group than the PBS group. Between D3 and D14, microvessel density increased significantly in the PB-MNC+ group, but it did not change significantly in the PB-MNC or PBS groups. Data are presented as mean ± SD. ^∗^*P* < 0.05. Scale bar = 400 *μ*m.

At D3, the number of Ki67+/CD31+ cells in the peri-infarct area was not significantly different between PB-MNC+, PB-MNC, or PBS-treated mice (76.4 ± 15.3, 55.3 ± 22.3 and 56.9 ± 15.9 cells, resp.) (*N* = 4) nor was microvessel density (14.4 ± 1.5 a.u., 15.0 ± 0.8 a.u., and 13.9 ± 0.4 a.u., resp.) (*N* = 4–5) (Figure 4). The levels of mRNA encoding for main proangiogenic factors were not significantly different between the PB-MNC+, PB-MNC, and PBS groups. Interestingly, we observed a significant increase in transforming growth factor (TGF)-*β* mRNA expression in PB-MNC+ compared to PBS-treated mice (*P* < 0.05) but no difference between PB-MNC, PBS, or PB-MNC+ groups. TGF-*β* receptor type-2 (TGFR-2) mRNA expression was not significantly different between the three groups (*N* = 5–8) (Table 2).

Ki67+/CD31+ cell quantification in the peri-infarct area was not performed on D14 because of the scarcity of Ki67+ cells in all three groups. We did however calculate microvessel density, which was significantly increased in the PB-MNC+ group compared to the PBS group (17.7 ± 2.0 a.u. versus 14.0 ± 0.9 a.u., *P* < 0.05) (*N* = 5–7); microvessel density in the PB-MNC group (15.8 ± 2.6 a.u.) was not significantly different to that of the other two groups (Figure 4). Levels of mRNA encoding Ang-1, Ang-2, Tie-2, eNOS, VEGF-A, VEGFR-2, PDGF-B, PDGFR-1, TGF-*β*, and TGFR-2 were not significantly different between the PB-MNC+, PB-MNC, or PBS groups (*N* = 5–7) (data not shown).

Between D3 and D14, microvessel density increased significantly in the PB-MNC+ group (*P* = 0.032) but was not significantly modified in the PB-MNC or PBS groups.

Plasma VEGF levels measured at D14 were significantly greater in mice treated with PB-MNC+ compared to the PBS group (73.5 ± 6.7 versus 41.6 ± 5.9 ng/L) (*P* < 0.001). There were however no significant differences between plasma VEGF levels in the PB-MNC group (55.7 ± 10.3 ng/L) and the other two groups (*N* = 6–7).

### 3.5. BBB Permeability

**Figure 5:**
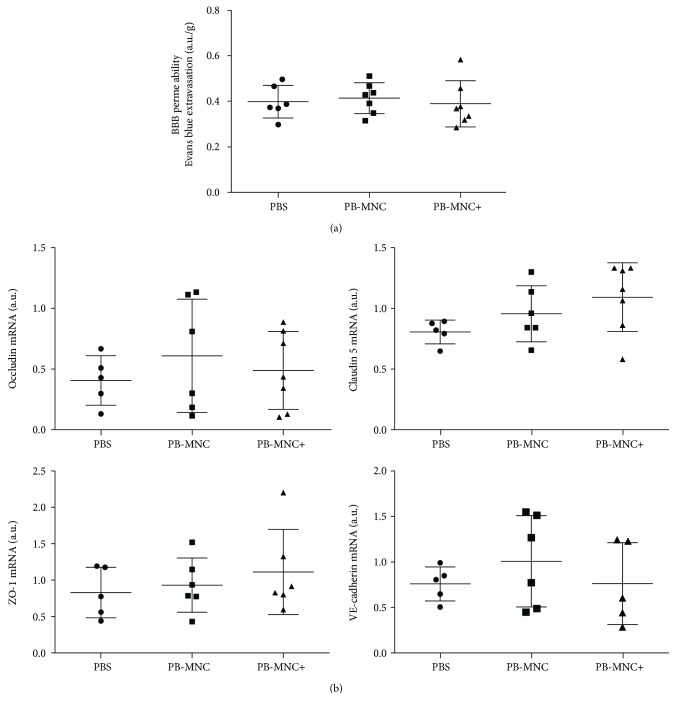
BBB permeability and expression levels of mRNA encoding BBB proteins. (a) PB-MNC, treated or not with ephrin-B2, compared to PBS, did not modify Evans blue extravasation (*N* = 6–7). (b) There was no significant difference in occludin, claudin 5, ZO-1, and VE-cadherin mRNA expression between the three groups of mice (*N* = 5–7). Data are presented as mean ± SD.

BBB permeability was assessed at D3 using Evans blue extravasation and by determining the expression levels of mRNA encoding BBB proteins. PB-MNC, stimulated or not with ephrin-B2, did not modify Evans blue extravasation (*N* = 6–7) or occludin, claudin-5, ZO-1, or VE-cadherin mRNA expression compared to PBS (*N* = 5–7) (Figure 5).

### 3.6. Inflammation

Inflammatory response was assessed at D3 and D14 by measuring microglia/macrophage cell density using Iba-1 immunostaining and by quantification of mRNA encoding proinflammatory cytokines interleukin-1*β* (IL-1*β*), IL-6, interferon-*γ* (IFN-*γ*), tumor necrosis factor-*α* (TNF-*α*), macrophage chemoattractant protein-1 (MCP-1), and the anti-inflammatory cytokine TGF-*β* and its receptor, TGFR-1.

**Figure 6:**
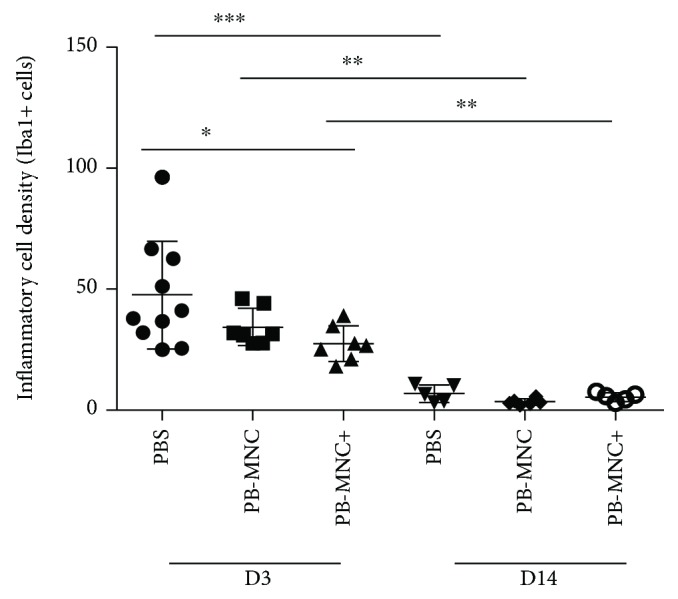
Microglia/macrophage cell density is decreased in mice treated with diabetic PB-MNC+. Iba-1 cell density was significantly decreased by ephrin-B2-stimulated PB-MNC compared to PBS at D3 (*N* = 7–10/group). By D14, microglia/macrophage density had significantly decreased in 3 groups compared to D3 with no inter groups differences (*N* = 5–6). Data are presented as mean ± SD. ^∗^*P* < 0.05; ^∗∗^*P* < 0.01; ^∗∗∗^*P* < 0.001.

At D3, there was an overall significant difference in microglia/macrophage density between the three groups (*P* = 0.043; Figure 6). In mice treated with PB-MNC+, Iba1+ cell density was significantly lower than in PBS-treated mice (27.4 ± 7.3 versus 47.4 ± 22.3) (*P* < 0.05); we observed no significant difference in Iba1+ cell density between the PB-MNC+ group compared to PB-MNC-treated mice (34.2 ± 7.6) (*N* = 7–10). We found no significant differences between PB-MNC+, PB-MNC, and PBS groups in terms of proinflammatory cytokines (Table 3). TGF-*β* mRNA expression was significantly greater in mice treated with PB-MNC+ than in PBS-treated mice *P* < 0.05 (Table 2).

At D14, microglia/macrophage density was not significantly different between the PB-MNC+, PB-MNC, and PBS groups (5.3 ± 1.7, 3.3 ± 1.2, and 6.8 ± 3.5 a.u., resp.; Figure 6) (*N* = 5–6), but had decreased dramatically compared to D3: PB-MNC+ (*P* = 0.0025), PB-MNC (*P* = 0.0012), and PBS (*P* = 0.0007). No intergroup differences in proinflammatory and anti-inflammatory cytokines were observed at D14, except for TNF-*α* mRNA expression, which was significantly higher in the PB-MNC+ group (1.4 ± 0.8 a.u.) than the PBS group (0.005 ± 0.006 a.u.) (*P* < 0.01), whereas TNF-*α* mRNA expression in the PB-MNC group (0.9 ± 0.6 a.u.) was not significantly different compared to the other two groups (*N* = 5–7).

### 3.7. Neurogenesis and Neuroblast Migration to the Infarct Area

Neurogenesis was assessed by calculating DCX-positive neuroblast density and by measuring the distance covered by these cells as they migrated from the SVZ to the peri-infarct area.

**Figure 7:**
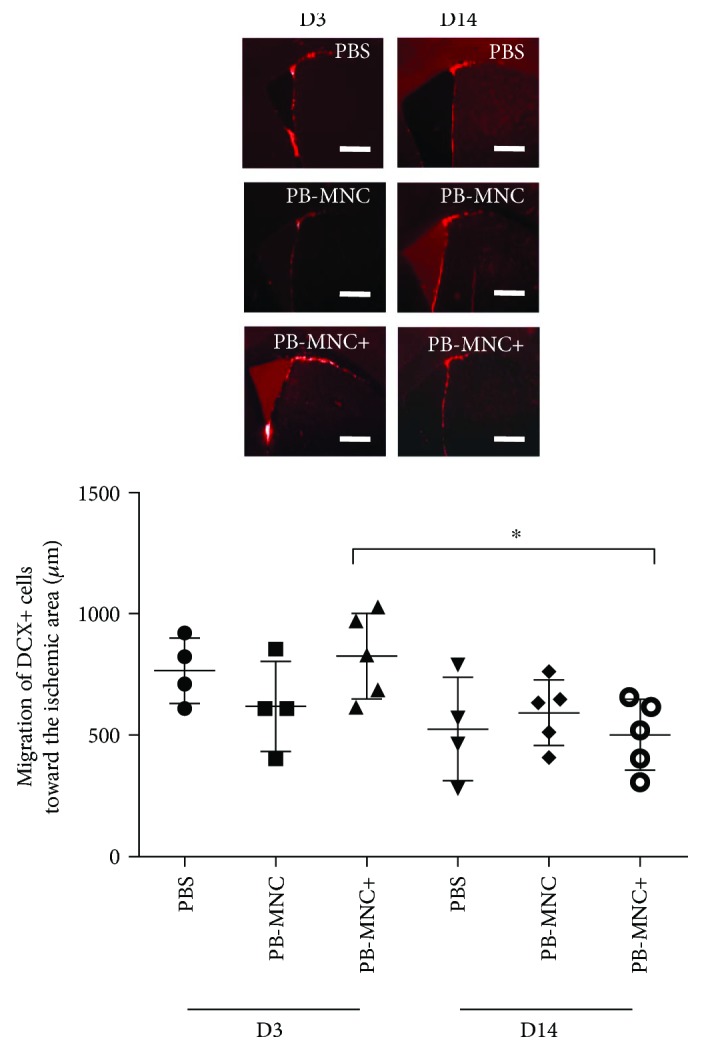
Neuroblast migration is not enhanced in mice treated with diabetic PB-MNC+. At D3 and at D14, there were no significant differences between the three groups in terms of the migration of DCX+ cells toward the ischemic area (*N* = 4–5/group). Between D3 and D14, there was a significant reduction in terms of neuroblast migration in PB-MNC+-treated mice. Data are presented as mean ± SD. ^∗^*P* < 0.05. Scale bar = 200 *μ*m.

At D3, there were no significant differences in neuroblast density between PB-MNC+, PB-MNC, and PBS groups (52,262 ± 14,825 *μ*m^2^; 47,579 ± 14,099 *μ*m^2^; and 57,225 ± 21,634 *μ*m^2^, resp.) or in the migration distance of DCX+ cells toward the ischemic area (825.6 ± 177.4 *μ*m; 618.8 ± 184.5 *μ*m; and 765 ± 135.5 *μ*m, resp.) (*N* = 4–5) (Figure 7).

Likewise, at D14, there were no significant differences in neuroblast density between the three groups (18,287 ± 5037 *μ*m^2^; 18,528 ± 9851 *μ*m^2^; and 12,476 ± 5223 *μ*m^2^, resp.) nor in the migration distance of DCX+ cells toward the peri-infarct area (500.5 ± 145.3 *μ*m; 592.3 ± 135.9 *μ*m; and 526.1 ± 212.5 *μ*m, resp.) (*N* = 4–5). Between D3 and D14, we observed a significant reduction in neuroblast density in the PB-MNC+ (*P* = 0.008), PB-MNC (*P* = 0.016), and PBS (*P* = 0.029) groups and a significant reduction in the neuroblast migration distance in the PB-MNC+ group (*P* = 0.032); no significant difference was observed between the same two time points in the PB-MNC and PBS groups.

BDNF, a major mediator of neuroplasticity, was also assessed; plasma BDNF levels measured at D14 were significantly greater in the PB-MNC+ groups (35.8 ± 3.7 ng/L) than the PB-MNC group (21.5 ± 1.4 ng/L) (*P* < 0.05) and the PBS group (20.5 ± 1.2 ng/L) (*P* < 0.01) (*N* = 6–7).

### 3.8. Cell Tracking

**Figure 8:**
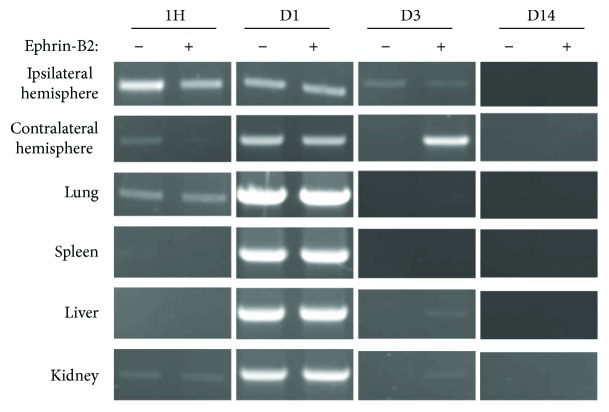
Cell tracking detection of human DNA in mice transplanted with human PB-MNC and PB-MNC+. DNA was extracted from mouse tissues and amplified using PCR to detect the human-specific P17H8 satellite sequence. Agarose gel analysis of PCR products demonstrated the presence of a 1171-bp human-specific fragment as early as one hour after cell injection mainly in the ipsilateral hemisphere and in the lungs of PB-MNC+ and unstimulated PB-MNC-treated mice. At D1 after injection, human DNA was readily detectable in the lungs, spleen, liver, and kidneys and in the contralateral hemisphere, whereas human DNA was still present in the ipsilateral hemisphere and had increased in the lungs. Signal disappeared from all organs, including brain, by D14 (*N* = 5).

PCR analysis revealed human genomic DNA in both the ipsilateral and contralateral hemispheres of mice injected with PB-MNC or PB-MNC+, as early as one hour postinjection and persisting one day later. One hour after injection, human DNA levels were lower in the contralateral hemispheres and (with the exception of the lungs) in the peripheral organs (the spleen, liver, and kidneys) than in the ischemic hemispheres; the signal increased however after one day, indicating that the engrafted cells had reached and were retained in the mouse brain (Figure 8). At D3, the signal was faint in the ischemic hemisphere, but was stronger in the contralateral hemisphere of mice treated with PB-MNC+ and was not detected in peripheral organs. At D14, no signal was detectable in either the brain or the peripheral organs.

### 3.9. The Subcellular Composition of PB-MNC Transplants and the PB-MNC Cellular Ephrin-B2/Fc-Binding Subsets

**Figure 9:**
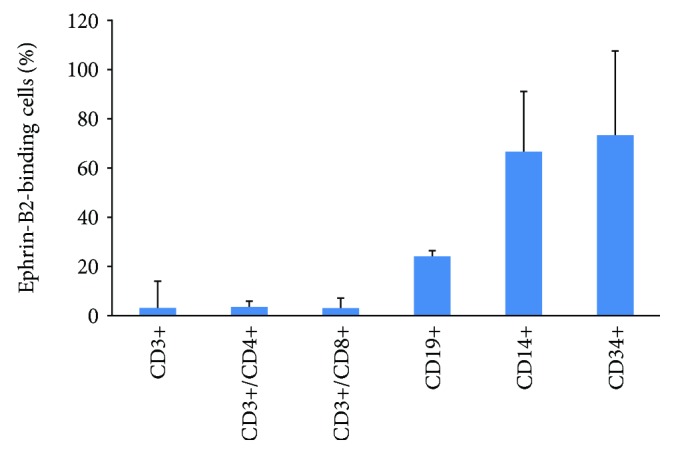
Percentage of PB-MNC subpopulations binding ephrin-B2/Fc (*N* = 5–6). Data are presented as mean ± SD.

PB-MNC isolated from diabetic patients (*N* = 8) were mainly composed of CD45+ cells (leucocytes) (97.5 ± 1.4%): CD3+ cells (T-lymphocytes) (64.6 ± 2.8%), CD3+/CD4+ cells (CD4+ T-lymphocytes) (43.1 ± 9.0%), CD3+/CD8+ cells (CD8+ T-lymphocytes) (19.2 ± 3.7%), CD19+ cells (B-lymphocytes) (6.6 ± 4.1%), CD14+ cells (monocytes) (15.8 ± 4.5%), and also for a small fraction of CD34+ cells (endothelial progenitor cells) (0.5 ± 0.3%). We have previously shown that only a fraction of 11.6 ± 11.6% cells within diabetic PB-MNC was capable to bind ephrin-B2/Fc [14]. Using multicolor flow cytometry analysis, we determined that the percentage of ephrin-B2/Fc-binding cells varied considerably, depending on PB-MNC subpopulation. The highest capacity to bind ephrin-B2/Fc was detected for CD34+ progenitors (73.3 ± 41.5%) and monocytes (66.6 ± 34.3%), while only a small proportion of T-lymphocytes, the largest fraction of PB-MNC, bound ephrin-B2/Fc (3.0 ± 2.4%). B-lymphocyte demonstrated an intermediate binding capacity (24.0 ± 24.5%) (*N* = 5–6) (Figure 9).

### 3.10. PB-MNC Response to Ephrin-B2 Stimulation by Microarray Analysis

**Figure 10:**
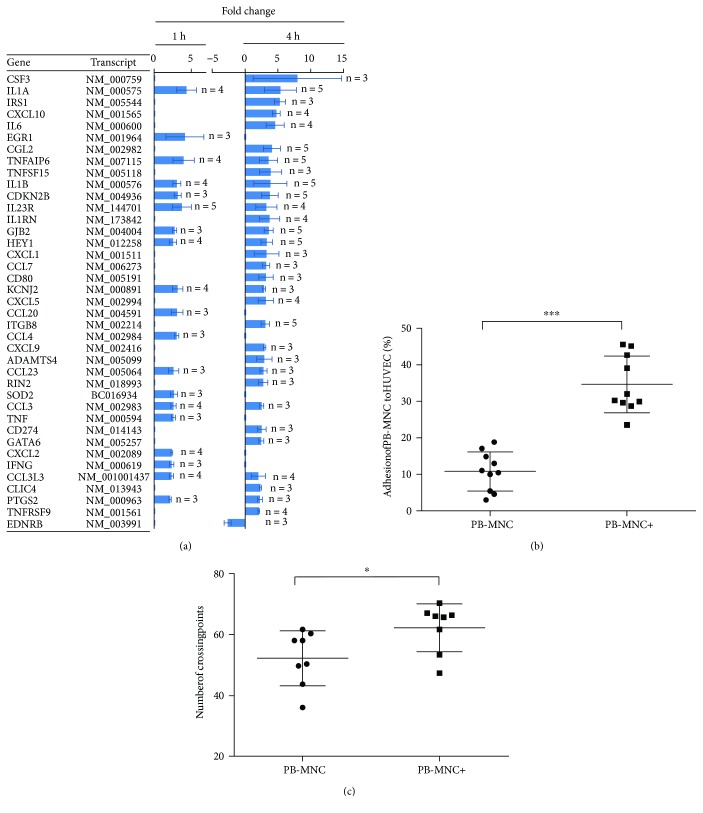
Ephrin-B2 stimulates the expression of genes involved in angiogenesis and inflammation of human diabetic PB-MNC. (a) Genes showing upregulated or downregulated expression after one hour (left graph) or four hours (right graph), in three or more of the total five samples per time point, are listed in descending order according to the average change in expression levels following ephrin-B2 stimulation. For a human gene to be considered significantly upregulated or downregulated by ephrin-B2, expression in the human array had to be at least twofold higher or lower, respectively. Figures to the right of the outer edge of bars indicate the number of patients in whom gene expression was significantly changed at both time points (*N* = 5). (b) Percentage adhesion of ephrin-B2-stimulated PB-MNC to HUVEC monolayer was increased 3.5-fold in adherent cells (*N* = 10/group). Data are presented as % adhesion ± HdSD. (c) Capillary-like tube formation assay on Matrigel. The number of branch points was significantly greater in the PB-MNC+ compared to PB-MNC groups (*N* = 7/group). Data are presented as mean ± SD. ^∗^*P* < 0.05; ^∗∗∗^*P* < 0.001.

To determine the mechanisms underlying the potentiating effect of ephrin-B2 on PB-MNC, we compared gene expression profiles of stimulated versus unstimulated cells, at 1 and 4 hours poststimulation, using Agilent Whole Human Genome Oligo Microarrays. At 1 hour, of the approximately 41,000 genes analyzed, the expression of 312 ± 101 genes was significantly changed, of which 90 ± 25 genes were downregulated and 223 ± 79 were upregulated more than twofold compared to unstimulated cells (*N* = 5) (Figure 10(a)).

At 4 hours, expression of an approximately 2.8-fold larger set of genes was modified, with expression profiles only partially overlapping with those obtained at 1 hour (total modulated 861 ± 496; downregulated 350 ± 208; upregulated 511 ± 307, *N* = 5). Of these genes, several were significantly upregulated or downregulated in three or more of the five patients (*P* < 0.0001), at one or both time points (Figure 10(a)). Among them, we found the principal components of major regulatory clusters, involving inflammatory responses (*IFNγ*, *IL6*, several members of the interleukin 1 pathway, including *IL1A*, *IL1B*, *IL1RN*, and the TNF pathway members *TNF*, *TNFAIP6*, *TNFSF15*, *TNFRSF9*), angiogenesis (*CXCL10*, *PTGS2*), immune regulation (*CD80*, *CD274*, *CSF3*, *IL23R*), leukocyte chemotaxis (*CCL2*, *CCL3*, *CCL4*, *CCL20*, *CCL23*, *CCL3L3*, *CXCL1*, *CXCL2*, *CXCL5*, *CXCL9*), transcriptional regulation (*EGR1*, *GATA6*, *HEY1*), proliferation (*CDKN2B*, *FGFR1*), adhesion (*ITGB8*), extracellular matrix remodeling (*ADAMTS4*), cell-cell communication (*GJB2*), cell signaling (*EDNRB*, *IRS1*, *RIN2*), ion transmembrane transport (*KCNJ2*, *CLIC4*), and response to oxidative stress (*SOD2*).

*IL23R* was upregulated in all tested patients one hour postephrin-B2 stimulation, and eight genes (*IL1A*, *IL1B*, *CCL2*, *CDKN2B*, *TNFAIP6*, *HEY1*, *ITGB8*, and *GJB2*) were upregulated in all patients four hours poststimulation. These may therefore be considered as potential gene candidates for activating the ephrin-B2 mechanism.

### 3.11. Adhesion of Ephrin-B2-Stimulated PB-MNC to HUVEC Monolayer

To assess the ability of PB-MNC+ to adhere to endothelial cells, we performed an *in vitro* adhesion assay. There was a 3.5-fold increase in the number of adherent cells with PB-MNC+ compared to PB-MNC (34.7 ± 7.8 versus 10.8 ± 5.3%, resp., *P* < 0.0001) (*N* = 10) (Figure 10(b)).

### 3.12. Capillary-Like Tube Formation Assay on Matrigel

To investigate the ability of human PB-MNC to stimulate angiogenesis *in vitro*, we performed an *in vitro* 3D Matrigel assay. Marked phenotypic differences in the capillary-like tubular patterns were observed between PB-MNC+/HUVEC and PB-MNC/HUVEC cocultures. Branch point density was significantly increased in the presence of PB-MNC+ compared to PB-MNC (62.2 ± 7.9 versus 52.2 ± 9.0, resp., *P* = 0.02) (*N* = 7), suggesting the formation of a more complex capillary-like network with greater covering capacity (Figure 10(c)).

## 4. Discussion

We speculated that PB-MNC from type-2 diabetic patients, stimulated by ephrin-B2 (PB-MNC+) and transplanted into mice 18–24 hours after induction of experimental cerebral ischemia, would be more effective than PBS or unstimulated PB-MNC in promoting stroke recovery. Our results show that IV transplantation of PB-MNC+ significantly reduces cerebral infarct volume and increases plasma BDNF levels compared to mice receiving PB-MNC or PBS. Compared to IV PBS, PB-MNC+ accelerated functional recovery and was associated with early cell proliferation in the SVZ and peri-infarct area, decreased microglia/macrophage cell density and TGF-*β* upregulation, and late angiogenesis. *In vitro* studies confirmed that ephrin-B2 modified the gene profile of PB-MNC, which acquired enhanced proangiogenic properties. Our data suggest that IV transplantation of ephrin-B2-stimulated PB-MNC from diabetic donors enhances brain repair.

Although over 400 trials (http://ClinicalTrials.gov web service) are currently investigating novel repair strategies for stroke patients, very little is known about ideal cell sources, total doses required, optimal administration routes, therapeutic windows, or even the mechanisms involved. We opted to adhere as closely as possible to the latest translational research, by exploring a strategy that favors PB-MNC transplantation, thus excluding cell culture expansion and any risk of graft rejection since patients receive autologous cell transplantation. The simplicity and rapidity of the ephrin-B2 treatment procedure (30 minutes) makes bedside cell injection feasible at any time. We also assessed the consequences of diabetes on the ability of cells to maintain their tissue repairing properties. The current pandemic of diabetes, a known cardiovascular risk factor associated with stroke, made this disease a particularly relevant context for research [21]. PB-MNC from diabetic patients differ (from healthy donor cells) in their phenotype and may therefore have exhausted their inherent aptitude to instigate neurogenesis and angiogenesis. It has been shown previously that diabetic endothelial progenitor cells exhibit impaired proliferation, adhesion, and incorporation in adhesion and matrigel assays [22] or, in vivo, cannot repair retina vasculature [23]. Actually, we did compare the effects induced by ephrin-B2 in PB-MNC isolated from healthy donors and from diabetic patients in our previous study [14 and unpublished data]. First, using the in vitro adhesion assay, we showed that PB-MNC from diabetic patients present a roughly 35% decreased ability to adhere to HUVEC, compared to healthy PB-MNC. Stimulation with ephrin-B2 resulted in restoration of impaired adhesion of diabetic PB-MNC so that the maximal response obtained at optimal time and concentration is comparable for diabetic PB-MNC and healthy PB-MNC. Secondly, using an in vivo hind limb ischemia model, we showed that the pretreatment with ephrin-B2 increased the therapeutic potential of PB-MNC isolated from healthy donors and from diabetic patients to a similar extent. Finally, we showed in our present study that mononuclear cells from peripheral blood of diabetic patients, and stimulated by ephrin-B2, are capable to enhance neurorepair in stroke.

Very few studies have assessed the efficacy of PB-MNC in the treatment of ischemic diseases. Experimental data have shown that cellular functions are reinforced through hypoxic preconditioning of human PB-MNC from healthy donors transplanted into a mouse hind limb model of ischemia [24]. Only one experimental model of cerebral hypoxia-ischemia has shown that transplantation of nonpreconditioned PB-MNC from healthy human donors reduced infarct volume and improved neurological deficit; brain recovery mechanisms were not specifically investigated [25]. Results from clinical studies using intracoronary transplantation of nonpreconditioned PB-MNC following acute myocardial infarction are however conflicting [26]. To the best of our knowledge, Broquères-You et al. [14] are the only group to have studied PB-MNC from diabetic donors and shown more extensive postischemic neovascularization when PB-MNC were stimulated by ephrin-B2. Our results also suggest that ephrin-B2 potentiated the neurorepair properties of PB-MNC from diabetic donors. Using microarray analysis, we showed that PB-MNC+ upregulate the genes that are commonly involved in proliferation, angiogenesis, and inflammation, processes all known to modulate neural plasticity [27]. *In vitro* analysis of PB-MNC adhesion to HUVEC monolayers, and also of capillary-like tube formation in coculture with HUVEC on Matrigel, confirmed the enhanced proangiogenic properties of PB-MNC+ compared to unstimulated PB-MNC. In our cerebral ischemia model, significantly more proliferative cells were observed in the peri-infarct area of the PB-MNC+ group as early as D3, followed by a significant increase in microvessel density and plasma VEGF-levels at D14, suggesting that angiogenesis was stimulated between D3 and D14 in that group. Angiogenesis is one of the mechanisms accounting for the neurorepair properties of BM-MNC [11], by promoting postischemic neurogenesis [12]. In our study, we found that early cell proliferation was induced by PB-MNC+ in the SVZ, a niche from which neuroblast progenitors are predominantly generated after stroke [12]. Moreover, plasma BDNF levels were increased in the PB-MNC+ group at D14. Because BDNF is known to have pleiotropic properties involved in neurorepair (angiogenesis, prevention of neuronal death, modulation of local inflammatory factors) in addition to neurogenesis and neural plasticity [27], the repair processes were likely stimulated by PB-MNC+ transplantation and persisted at D14. However, intergroup DCX+ cell density differences were not significant, on D3 or on D14. An earlier neuron progenitor marker such as glial fibrillary acidic protein (GFAP) or Mash1 and an intermediate time point analysis may have been more appropriate to detect neurogenesis in the mice treated with PB-MNC+ [28]. Furthermore, we could not show either that transplantation with PB-MNC+ increased the distance covered by neuroblasts to the infarct area at D14, on the contrary. We can speculate that PB-MNC+ may have accelerated neuroblast differentiation into neurons, mature neurons do not expressing anymore DCX.

Evans blue extravasation and BBB gene expression assessments showed that BBB permeability was not modified by treatment. TGF-*β* mRNA however, which has a role in vessel maturation and stability [29], was significantly increased in mice treated with PB-MNC+. Moreover, the results of our microarray analysis revealed the upregulation of several gene products linked to TGF-*β* signaling, including integrin *β*8 that has been implicated in both TGF-*β*-mediated inflammation suppression [30] and stabilization of nascent brain vessels [31, 32], as well as IL-1*β* [32] and Hey1 [33]. Thus, the increased expression of TGF-*β* mRNA at D3 may also be a marker of an anti-inflammatory response. Indeed, microglia/macrophage cell density was lower in the PB-MNC+ than the PBS group, suggesting that PB-MNC+ may limit the early excitotoxic cascade triggered by ischemia and exert a cerebroprotective effect, as was previously shown following intranasal administration of TGF-*β* in mice poststroke [34], and therefore have contributed to the significant reduction in infarct volume that we observed. Although we did not specifically investigate potential sources of TGF-*β*, astrocytes are known to have an anti-inflammatory effect in the subacute poststroke period and are a major source of TGF-*β* [35] so may well be good candidates.

At D14, TNF-*α* was significantly increased in the PB-MNC+ group. The role of TNF-*α* in stroke is controversial and either deleterious or beneficial in stroke, depending on the local source of production (microglia, lymphocytes, astrocytes, or neurons), the timing poststroke, and the specific receptor subtype that is activated [36]. A dedicated study to elucidate these points is warranted.

The decreased microglia/macrophage density following PB-MNC+ treatment, and the absence of upregulation of proinflammatory cytokines, suggests that human cell transplantation did not induce rejection in our immunocompetent recipients. One hour after injection, we detected human DNA in the ischemic brain hemisphere and the lungs; 24 hours later, it had increased in the contralateral hemisphere and in all peripheral organs collected, including the lungs. The signal persisted in the contralateral hemisphere of PB-MNC+ transplanted mice on D3 and to a lesser extent in the ipsilateral hemisphere in both PB-MNC and PB-MNC+ groups, but had totally disappeared by D14. The absence of notable differences between PB-MNC and PB-MNC+ suggests that PB-MNC recruitment was not significantly modified by ephrin-B2 stimulation, except at D3 in the contralateral hemisphere; this requires confirmation in a larger mouse population.

Taking into account the higher ephrin-B2-binding efficiency of monocytes and CD34+ progenitors, we hypothesize that these cellular subpopulations represent the principal ephrin-B2 targets within PB-MNC and contribute for a major part to the therapeutic effects of ephrin-B2-treated PB-MNC. The recent experimental findings on the role of monocytes in therapeutic efficacy of MNC isolated from umbilical cord blood (UCB-MNC) [37] and the contribution of CD34+ cells to the neuroprotective effect of UCB-MNC [38] support our hypothesis.

Our study has several limitations. First, we evaluated one single-cell dose, though it was the highest tolerated dose determined in our previous experiments as inducing the maximal proangiogenic effect [14]. Second, defining optimal timing of the injection is a genuine challenge. Our study design included a single injection at what is currently the most frequently studied injection time point, that is, not immediately poststroke, because of the deleterious excitotoxic cascade, but not too late, particularly if both neuroprotection and neurorepair are targeted. Third, our assessment time points may have been too far apart to identify a specific mechanism. This might account for our inability to demonstrate neurogenesis or to show significant differences between PB-MNC+ and PB-MNC-treated mice for some objectives. Fourth, the known rapid recovery of C57Bl6J mice subjected to pMCAo, particularly when the infarct is small, prevented us from showing any significant differences between PB-MNC+ and PB-MNC-treated mice on the neurological score assessment or the ink test. Fifth, we did not show any difference in glial scar or prevention of brain atrophy at day 14; as it might occur later in the time course, it could be an interesting point to look at as well as prevention of cognitive decline, but this would deserve a specific study. Lastly, the use of animals with no vascular risk factors may have overestimated the potential of the therapeutic strategies we tested [39]. There is a paucity of data on the host environment in the context of cell transplantation. We do know that diabetic microangiopathy has the potential to interfere with repair mechanisms [29] and the cerebral “diabetic state” may generate inflammation that affects the global response to cell transplantation. BM-MNC have been tested in older mice and found to be beneficial in this population [40], though not in spontaneously hypertensive rats [4, 41]. Transplantation in diabetic mice should be the next step in the development of such cell therapy strategies. Moreover, although this was not our objective, the question of the ideal therapeutic window remains open. In a comparable study using intravenous administration of human umbilical cord blood mononuclear cells at 4, 24, and 72 hours and 14 or 120 days after permanent middle cerebral artery occlusion in spontaneously hypertensive rats, it was shown that the administration was effective up to 72 hours after stroke, which enlarges the window compared to recanalization strategies [42]. Whether this is also the case in mice transplanted with human PB-MNC demands a specific study.

We have shown for the first time that IV transplantation of PB-MNC+ from diabetic donors, 18–24 hours after mouse cerebral ischemia, significantly reduces infarct volume, enhances cell proliferation and angiogenesis, and modulates inflammation. We have also shown that unstimulated PB-MNC were not significantly more effective on these endpoints than PBS, despite a trend towards improvement. Finally, we have shown that diabetic human PB-MNC, when stimulated by ephrin-B2, retain their neurorepair properties when transplanted poststroke. This strategy allowed us to assess the beneficial effect of MNC stimulated by ephrin-B2 in a nondiabetic ischemic brain. In line with current translational research, future studies should explore (i) whether the diabetic state of the recipient influences the response to cell transplantation and (ii) whether PB-MNC collected from diabetic patients in the acute poststroke period maintain the same aptitude since stroke is known to influence the systemic inflammatory response and bone marrow activation. Focus should be given to the gene candidates identified in our study since triggering the TGF-*β* pathway may underlie the ephrin-B2-mediated therapeutic poststroke effects. BM-MNC and PB-MNC should also be compared to assess the efficacy of the source.

## Figures and Tables

**Table 1:** Sense and antisense primer sequences used for RT-PCR.

Name	Sense	Antisense
Occludin	CCTCCAATGGCAAAGTGAAT	CCCCACCTGTCGTGTAGTCT
Claudin 5	GCTCTCAGAGTCCGTTGACC	ATCTAGTGCCCCCAGGATCT
TJP-1 gene (ZO-1 protein)	ACCCAGCAAAGGTGTACAGG	CCGTAGGCGATGGTCATAGT
VE-cadherin	CAATGACAACTTCCCCGTCT	CGTTTGGGGTCTGTCTCAAT
Angiopoietin 1	GGTCAACAGAATCGCCACTT	CCTGTTCCCATTTGCTGTTT
Angiopoietin 2	AATGTTCCGTGGGAGTTCAG	AACCTGTGCCCACCACTTAG
Tie-2 (or TEK)	TCTGGGTGGCCACTACCTAC	TGAAAGGCTTTTCCACCATC
eNOS	AAGCTGCAGGTATTTGATGC	TATAGCCCGCATAGC
VEGF-A	CCTTAATCCAGAAAGCCTGACATG	AAAGTGCTCCTCGAAGAGTCTCC
VEGFR-2	GGCGGTGGTGACAGTATCTT	GTCACTGACAGAGGCGATGA
PDGF-B	GGCCACACACCTTCTCTGAT	GTGGAGGAGCAGACTGAAGG
PDGFR-1	CACCTTCTCCAGTGTGCTGA	GGAGTCCATAGGGAGGAAGC
TGF-*β* (1)	TTGCTTCAGCTCCACAGAGA	TGGTTGTAGAGGGCAAGGAC
TGFR-2	GCAAGTTTTGCGATGTGAGA	GGCATCTTCCAGAGTGAAGC
Interleukin-1*β*	ACCTTCCAGGATGAGGACATGA	AACGTCACACACCAGCAGGTTA
Interleukin-6	AGTTGCCTTCTTGGGACTGA	TCCACGATTTCCCAGAGAAC
Interferon-*γ*	GCTTTGCAGCTCTTCCTCAT	GTCACCATCCTTTTGCCAGT
TNF-*α*	TGGCCTCCCTCTCATCAGTTC	TTGGTGGTTTGCTACGACGTG
MCP-1 (or CCL2)	AGGTCCCTGTCATGCTTCTG	TCTGGACCCATTCCTTCTTG

TJP-1: tight junction protein 1; ZO-1: zona occludens-1; VE-cadherin: vascular endothelial-cadherin; eNOS: endothelial nitric oxide synthase; VEGF-a: vascular endothelial growth factor-a; VEGF-R2: vascular endothelial growth factor receptor 2; PDGF-B: platelet-derived growth factor-B; PDGFR-1: platelet-derived growth factor receptor-1; TGF-*β*: transforming growth factor-*β*; TGFR-2: transforming growth factor-*β* receptor-2; TNF-*α*: tumor necrosis factor-*α*; MCP-1: monocyte chemoattractant protein-1.

**Table 2:** Relative proangiogenic factor transcript levels in treated and untreated mice at D3 (*N* = 5–8).

Transcript	PBS	PB-MNC	PB-MNC+	*P* values
Ang-1	0.7 ± 0.4	0.9 ± 0.3	1.0 ± 0.3	0.42
Ang-2	0.8 ± 0.4	0.9 ± 0.5	0.9 ± 0.6	0.99
Tie 2	1.2 ± 0.4	1.2 ± 0.6	1.8 ± 1.4	0.84
eNOS	0.5 ± 1.6	0.9 ± 0.6	1.0 ± 0.6	0.40
VEGF-A	0.6 ± 1.2	0.9 ± 0.6	0.9 ± 0.8	0.79
VEGFR-2	0.7 ± 0.3	0.9 ± 0.5	1.0 ± 0.4	0.55
PDGF-B	2.5 ± 1.3	2.6 ± 0.6	2.4 ± 0.6	0.75
PDGFR-1	0.6 ± 0.2	1.0 ± 0.3	1.0 ± 0.6	0.07
TGF-*β*	0.7 ± 0.3	0.9 ± 0.3	1.2 ± 0.4	*P* < 0.05
TGFR-2	3.7 ± 0.4	4.3 ± 1.6	3.2 ± 1.3	0.62

Ang-1: angiopoietin-1; Ang-2: angiopoietin-2; Tie-2: angiopoietin tyrosine kinase receptor; eNOS: endothelial nitric oxide synthase; VEGF-A: vascular endothelial factor-A; VEGFR-2: VEGF receptor-2; PDGF-B: platelet-derived growth factor-B; PDGFR-1: PDGF receptor-1; TGF-*β*: transforming growth factor-*β*; TGFR-2: transforming growth factor-*β* receptor-2. Data are presented as mean (arbitrary units) ± SD.

**Table 3:** Relative proinflammatory cytokines transcript levels in treated and untreated mice at D3 (*N* = 5–7).

Transcript	PBS	PB-MNC	PB-MNC+	*P* values
IL-1 *β*	1.9 ± 1.0	5.4 ± 5.2	4.0 ± 3.6	0.37
IL-6	1.0 ± 0.4	1.4 ± 0.3	2.1 ± 1.4	0.15
IFN-*γ*	2.5 ± 2.5	2.0 ± 1.5	2.1 ± 1.1	0.89
TNF-*α*	0.4 ± 0.2	0.6 ± 0.3	0.8 ± 0.8	0.60
MCP-1	0.7 ± 0.5	1.0 ± 0.5	1.4 ± 1.0	0.39

IL-1*β*: interleukin-1*β*; IL-6: interleukin-6; IFN-*γ*: interferon-*γ*; TNF-*α*: tumor necrosis factor-*α*; MCP-1: macrophage chemoattractant protein-1. Data are presented as mean (arbitrary units) ± SD.
